# Dual block evidence of the effects of topiramate, a sulfamate-substituted monosaccharide, on voltage-gated sodium current and hyperpolarization-activated cation current

**DOI:** 10.1186/s40360-025-01043-6

**Published:** 2025-11-29

**Authors:** Ray-Chang Tzeng, Ming-Chi Lai, Sheng-Nan Wu, Chin-Wei Huang

**Affiliations:** 1 Department of Neurology, Tainan Municipal Hospital (Managed by Show Chwan Medical Care Corporation), Tainan, Taiwan; 2 Department of Pediatrics, Chi-Mei Medical Center, Tainan, Taiwan; 3 Department of Research and Education, An Nan Hospital, China Medical University, Tainan, Taiwan; 4 School of Medicine, National Sun Yat-sen University College of Medicine, Kaohsiung, Taiwan; 5 Department of Neurology, National Cheng Kung University Hospital, College of Medicine, National Cheng Kung University, Tainan, Taiwan

**Keywords:** Topiramate, Voltage-gated Na^+^ current, Late Na^+^ current, Transient Na^+^ current, Hyperpolarization-activated cation current

## Abstract

**Background:**

Topiramate (TPM) is a sulfamate-substituted monosaccharide known for its wide-ranging effects on epilepsy, neuropathic pain, and migraines. However, its precise influence on plasmalemmal ionic currents, including their magnitude and gating kinetics, remains uncertain. Therefore, a reassessment of the regulatory effect of TPM on ionic currents in electrically excitable cells is warranted.

**Methods:**

With the aid of patch clamp technology, we investigated the effects of TPM on the amplitude, gating, and hysteresis of plasmalemmal ionic currents from GH_3_ lactotrophs.

**Results:**

We observed that TPM exhibited a concentration-dependent inhibition of both transient (*I*_Na(T)_) and late (*I*_Na(L)_) components of *I*_Na_, activated by brief depolarizing stimuli. At low concentration, TPM did not show any noticeable effect on *I*_Na(T)_; however, it was effective in reducing *I*_Na(L)_ amplitude. TPM caused a leftward shift in the midpoint of the steady-state inactivation curve of *I*_Na(T)_ without altering the gating charge. Importantly, the overall current density versus voltage relationship of *I*_Na(T)_ remained unaltered during TPM exposure. Intriguingly, the reduction in *I*_Na(T)_ induced by TPM could not be reversed by subsequent additions of flumazenil or chlorotoxin. Furthermore, TPM suppressed the density of the hyperpolarization-activated cation current (*I*_h_). Simultaneously, the activation time course of *I*_h_ slowed in the presence of TPM. Moreover, TPM exposure decreased the hysteretic strength activated by double triangular ramp voltage, a change partially reversed by oxaliplatin. In current-clamp potential recordings, spontaneous action potentials were susceptible to suppression in the presence of TPM.

**Conclusions:**

Collectively, these findings strongly suggest that TPM’s effects on *I*_Na_ and *I*_h_ have the potential to impact the functional activities and electrical behaviors of excitable cells.

## Background

Topiramate (TPM) is an oral or intravenous medication used to prevent or reduce epileptic seizures. It can be used alone or in combination with other therapies in neuropathic or migraine pain. This drug is a sulfamate-substituted monosaccharide and several studies suggest that TPM may act as a modulator of γ-aminobutyric acid type A (GABA_A_) receptors [1–6]. Studies on endocrinology have reported that TPM can help maintain metabolism-secretion coupling in insulin-secreting cells [7–9]. However, the administration of TPM has been associated with negative side effects that have been gradually observed [10, 11]. Additionally, TPM has been proposed as a potential treatment for antipsychotic-induced hyperprolactinemia [12, 13]. Therefore, it is important to reevaluate the underlying mechanisms of action of TPM in excitable cells, especially neuroendocrine cells.

There are nine isoforms of voltage-gated Na^+^ (Na_V_) channels, which are commonly referred to as Na_V_1.1-1.9 (or SCN1A-SCN5A and SCN8A and SCN11A). These channels are widely distributed in mammalian excitable tissues, including the neuroendocrine or endocrine systems, as well as the central or peripheral nervous systems [14, 15]. When rapidly activated, Na_V_ channels generate the whole-cell voltage-gated Na^+^ current (*I*_Na_), which depolarizes the surface membrane and initiates, generates, and propagates action potentials (APs). Changes in the magnitude and gating of *I*_Na_ can modulate the firing amplitude, frequency, and discharge patterns inherent in different excitable cells [14, 16]. Na_V_ channel antagonists have been reported to be useful for the treatment of migraine [17, 18]. However, it is currently unclear whether cell exposure to TPM can modify the magnitude and gating kinetics of *I*_Na_, although a few papers have demonstrated the ability of TPM to suppress *I*_Na_ [19, 20].

The hyperpolarization-activated cation current (*I*_h_ or funny current [*I*_f_]) is a well-known regulator of repetitive electrical activity in various neuron types, as well as neuroendocrine, endocrine, and heart cells [21–28]. *I*_h_ is a unique inward-rectifying current that can conduct both Na^+^ and K^+^ ions. Its activity can be inhibited by compounds like CsCl, ivabradine, or ZD7288 [23, 25, 26]. Activation of *I*_h_ occurs at the resting membrane potential, leading to a predominant inward current carried by Na^+^ ions. This depolarizes the membrane and plays a crucial role in modulating the rhythmic firing of action potentials (APs) [23, 25]. *I*_h_ is mediated by channels encoded by the hyperpolarization-activated cyclic nucleotide-gated (HCN) gene family, which belongs to the superfamily of voltage-gated K^+^ (K_V_) channels and cyclic nucleotide-gated (CNG) channels.

The HCN2, HCN3, or mixed HCN2 + HCN3 channels have previously been reported to be present in GH_3_ lactotrophs [23, 24]. Recent research has suggested a strong connection between the activity of HCN channels and migraine [29–32]. Additionally, the functioning of CNG and HCN channels is thought to be related to phototransduction in photosensitive retinal ganglion cells [33]. However, it remains unclear how TPM can interact with the HCNx channels to alter the magnitude, gating kinetics, and hysteretic behavior of *I*_h_.

Taking into account the aforementioned factors, the objective of this study was to explore the possible electrophysiological effects of TPM on pituitary GH_3_ cells. In particular, we investigated how TPM influences the amplitude and gating properties of *I*_Na_ and *I*_h_ currents. Our findings indicate that these ionic currents, specifically *I*_Na_ and *I*_h_, could serve as a unique and specific target for TPM-mediated modulation of cellular function.

## Methods

### Chemicals, drugs, reagents, and solutions utilized in this work

Sigma-Aldrich (Genechain, Kaohsiung, Taiwan) provided the following substances: TPM (topiramic acid; Topamax^®^, Epitomax^®^, 2,3:4,5-bis-O-(1-methylethylidene)-β-D-fructopyranose sulfamate; [(1*R*,2*S*,6*S*,9*R*)-4,4,11,11-tetramethyl-3,5,7,10,12-pentaoxatricyclo[7.3.0.0^2,6^]dodecan-6-yl]methyl sulfamate, C_12_H_21_NO_8_S, CAS No.: 97240-79-4), flumazenil (FLM, CAS No.: 78755-81-4), oxaliplatin (OXAL, CAS No.: 61825-94-3), tetraethylammonium chloride (TEA, CAS No.: 56-34-8), and tetrodotoxin (TTX, Product No.: 554412). Chlorotoxin was provided by the laboratory of Professor Dr. Woei-Jer Chuang from the Department of Biochemistry at National Cheng Kung University College of Medicine in Tainan, Taiwan. Culture media, fetal calf serum, horse serum, L-glutamine, and trypsin/EDTA were provided by HyClone™ (Genechain), while all other reagents or chemicals utilized were sourced from reputable suppliers to ensure the highest purity.

The bath solution used in this study, which was a normal Tyrode’s solution, had the following composition: 136.5 mM NaCl, 1.8 mM CaCl_2_, 5.4 mM KCl, 0.53 mM MgCl_2_, 5.5 mM glucose, and 5.5 mM HEPES-NaOH buffer, pH 7.4. To record whole-cell *I*_h_ or membrane potential, the measuring electrodes were backfilled with a solution composed of 130 mM K-aspartate, 20 mM KCl, 1 mM KH_2_PO_4_, 3 mM Na_2_ATP, 0.1 mM Na_2_GTP, 0.1 mM EGTA, and 5 mM HEPES-KOH buffer, pH 7.2. To prevent potential contamination of Cl^−^ currents, Cl^−^ ions in the pipette internal solution were replaced with aspartate. For the recording of voltage-gated Na^+^ current (*I*_Na_), K^+^ ions in the pipette internal solution were replaced with equimolar Cs^+^ ions, and the pH was adjusted to 7.2 using CsOH. Both the filling and bathing solutions, as well as the culture media, were filtered using sterile Acrodisc syringe filters containing a 0.2-µm Supor Membrane (Bio-Check; New Taipei City, Taiwan).

### Cell culture

The GH_3_ pituitary adenomatous cell line (BCRC-60015) was obtained from the Bioresource Collection and Research Center in Hsinchu, Taiwan. The cells were cultured in Ham’s F medium supplemented with 2.5% (*v/v*) newborn calf serum, 15% (*v/v*) horse serum, and 2 mM L-glutamine. They were grown in a monolayer culture at 37 °C in a humidified environment of 1:19 carbon dioxide/air. The culture medium was typically changed every 2–3 days. Electrophysiological measurements were conducted five to six days following the attainment of 60–80% confluence by the cells.

### Patch-clamp technique used for electrophysiological measurements

Just before recordings, we gently dissociated GH_3_ cells using a 1% trypsin-EDTA solution, and then added a few drops of the resulting cell suspension containing cell clumps into a custom-built chamber. The chamber was tightly mounted on the working stage of a DM-II inverted phase-contrast microscope (Leica; Major Instruments, Tainan, Taiwan). The cells were kept at room temperature (20–25 °C) and immersed in HEPES-buffered normal Tyrode’s solution with an ionic composition as previously mentioned. The electrodes were fabricated from Kimax^®^-51 soft-glass capillaries with an outer diameter of 1.5–1.8 mm (#34500-99; Kimble; Dogger, New Taipei City, Taiwan) using a Narishige PC-100 vertical puller (Sunpoint, Taoyuan, Taiwan). The tips of the electrodes were fire-polished with a microforge (MF-83; Narishige, Sunpoint) before being filled with the various internal solutions described above. The measured resistances of the electrodes were 3–5 MΩ. Once inserted into a holder, the electrode was maneuvered using a WR-98 micromanipulator (Narishige, Sunpoint). We used a modified patch-clamp technique to record ionic currents or membrane potential, with the whole-cell current or potential recordings, respectively, using an RK-400 patch amplifier (Bio-Logic, Claix, France) connected to a laptop computer. The methodology has been previously described [16, 23, 26]. Before establishing the GΩ-seal, we commonly corrected for a liquid junction potential (about − 13 mV) that developed at the electrode’s tip due to the difference in the compositions of the internal and bath solution and then corrected the whole-cell data accordingly. During measurements, we used a homemade gravity-driven bath perfusion system to exchange the solution. To measure the *I*_h_, we clamped the examined cell at a holding potential of − 40 mV before a long-lasting hyperpolarizing pulse was applied.

The amplified signal output, including both potential and current traces, was continuously monitored at fixed intervals and stored digitally online at a sampling rate of ≥ 10 kHz using an ASUS-ExpertBook laptop computer (Yuan-Dai, Tainan, Taiwan). To facilitate the analog-to-digital (A/D) and digital-to-analog (D/A) conversion during measurements, we used a low-noise Digidata^®^ 1440 A digitizer, which was connected to an ASUS computer and operated using pClamp™ 10.6 software run under Microsoft Windows 7 (Redmond, WA). To minimize noise and other artifacts, we applied an FL-4 four-pole Bessel filter (Dagan, Minneapolis, MN) to low-pass filter current signals at 2 kHz. The voltage-clamp protocols with rectangular or ramp waveforms were designed using pClamp software and applied to the tested cells through D/A conversion. These protocols were used to assess the current density versus voltage relationship as well as the steady-state activation and inactivation curves of the current being measured.

### Analyses of whole-cell data

To investigate the concentration-dependent inhibition of TPM on the density of *I*_Na(T)_, *I*_Na(L)_, and *I*_h_, GH_3_ cells were kept in Ca^2+^-free, Tyrode’s solution. *I*_Na(T)_ and *I*_Na(L)_ were measured by depolarizing the tested cells from − 100 to − 10 mV for a duration of 40 ms, with *I*_Na(T)_ and *I*_Na(L)_ recorded at the beginning and end-pulse of step depolarization, respectively. For recording *I*_h_, each cell was clamped at a holding potential of − 40 mV, and a long-lasting step hyperpolarization to − 110 mV for 2 s was applied to evoke the slowly activating *I*_h_. The amplitude of *I*_Na(T)_, *I*_Na(L)_, or *I*_h_ was then measured during cell exposure to different RPM concentrations. The observed concentration-dependent decrease of *I*_Na(T)_, *I*_Na(L)_, or *I*_h_ by RPM in GH_3_ cells was determined by fitting the data to a Hill function. That is,

$${\text{percentagedecrease}}\left({\text{\% }} \right) = \frac{{({{\left[{TPM} \right]}^{{n_H}}} \times {E_{max}})}}{{(IC_{50}^{{n_H}} + {{\left[ {TPM} \right]}^{{n_H}}})}}$$

where [RPM] represents the TPM concentration applied to the tested cell, n_H_ is the Hill coefficient which indicates the level of cooperativity in the inhibition process, IC_50_ is the concentration of TPM required to produce 50% inhibition of the current (*I*_Na(T)_, *I*_Na(L)_, or *I*_h_), and *E*_max_ represents the maximal inhibition of the specified current induced by TPM.

To examine the impact of 10 µM TPM on the quasi-steady-state activation or inactivation of *I*_Na(T)_, we generated plots illustrating the normalized conductance or density of *I*_Na(T)_ against the membrane or conditioning potential, both in the presence and absence of 10 µM TPM. Subsequently, we applied the Boltzmann equation [34] to fit the acquired dataset. The equation is expressed as:

$$\begin{gathered} \:\raisebox{1ex}{$G$}\!\left/\:\!\raisebox{-1ex}{${G}_{max}$}\right.or\:\raisebox{1ex}{$I$}\!\left/\:\!\raisebox{-1ex}{${I}_{max}$}\right.\cr \qquad\qquad\qquad\qquad\qquad\qquad=\raisebox{1ex}{$1$}\!\left/\:\!\raisebox{-1ex}{$\left\{1+exp\left\lfloor\pm\:\left(V-{V}_{1/2}\right)RT/qF\right\rfloor\right\}$}\right.\end{gathered}$$

In this expression, *G*_max_ or *I*_max_ represents the maximal conductance or current density of *I*_Na(T)_ measured in the absence and presence of TPM. The variable *V* denotes the membrane or conditioning potential in millivolts (mV), while *V*_1/2_ is the voltage at which half-maximal activation or inactivation occurs—commonly referred to as the midpoint potential. The parameter *q* is the apparent gating charge, expressed in units of the elementary charge (*e*), *F* is the Faraday constant, *R* is the universal gas constant, and *T* is the absolute temperature.

The Boltzmann equation plays a crucial role in describing the relationship between the membrane potential and the probability of ion channel gating (such as activation or inactivation) in the steady state, particularly for sodium (Na^+^) channels. The Boltzmann equation can helps evaluate key parameters like V₁/₂ (the membrane potential at which 50% of the channels are either open or inactivated) and gating charge (which reflects the movement of charge across the membrane as the channel transitions between states).

To determine the conductance (*G*) value of *I*_Na(T)_, current density was divided by the difference between membrane potential and the reversal potential of *I*_Na(T)_ (i.e., + 50 mV).

For determination of inactivation time constant, data points representing the inactivation time course of *I*_Na_, both with or without the addition of TPM, were fitted to a biexponential function, i.e.,

$$\:\text{y}=\text{A}\times\:\left({e}^{\frac{-t}{{\tau\:}_{fast}}}\right)+B\times\:\left({e}^{\frac{-t}{{\tau\:}_{slow}}}\right)$$

where y is the relative amplitude of *I*_Na_ at time t, A or B is the relative amplitude of each exponential component, and τ_fast_ or τ_slow_ is the fast or slow time constant in the time course of *I*_Na_ inactivation, respectively.

### Curve-fitting methods and statistical analyses

Curve-fitting analyses, including both linear and nonlinear methods (e.g., Boltzmann function or one- and two-exponential curves), were conducted using various analytical tools such as the Microsoft Excel^®^ 2019 “Solver” add-in with VBA programming support and the OriginPro^®^ 2021 software from Microcal (Scientific Formosa, Kaohsiung, Taiwan), utilizing least-squares minimization techniques. The mean ± standard error of the mean (SEM) is presented for the average data (i.e., whole-cell current or potential data), and the number of independent samples (n) indicates the number of cells used for data collection. Data distribution was assessed through normality tests. Between-group comparisons employed either paired or unpaired Student’s *t*-test, while more than two groups were compared using one-way or two-way analysis of variance (ANOVA) with or without repeated measures. Post-hoc Fischer’s least-significant difference (LSD) test was conducted for further analysis. Statistical analyses were carried out using IBM SPSS 23.0 (AsiaAnalytics, Taipei, Taiwan). Statistical significance was set at *p* < 0.05, indicated by ^*^, Ɨ, or ^#^ in the figures.

## Results

### Inhibitory effect of topiramate (TPM) on voltage-gated Na^+^ current (I_Na_)

**Fig. 1:**
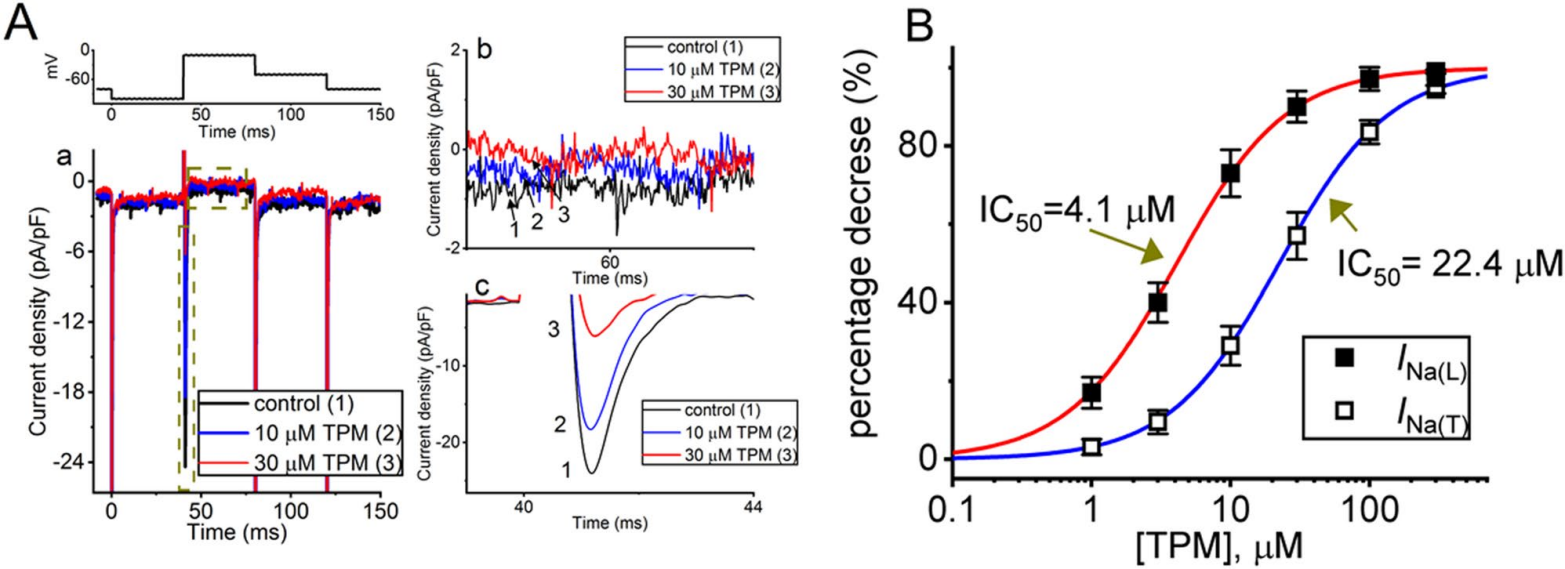
Effect of topiramate (TPM) on voltage-gated Na^+^ current (*I*_Na_) in pituitary GH_3_ cells. **(A)** Representative current traces obtained during the control period (labeled 1, black color) and after cell exposure to 10 µM TPM (labeled 2, blue color) or 30 µM TPM (labeled 3, red color). The extracellular solution was Ca^2+^-free, Tyrode’s solution containing 10 mM tetraethylammonium chloride (TEA) and 0.5 mM CdCl_2_, while the recording electrode was backfilled with a Cs^+^-enriched solution. Panel b or c on the right side, respectively, shows the expanded current traces in the dashed box with the curve arrow of panel a at the left side. **(B)** Concentration-response curve of TPM-induced inhibition of *I*_Na(T)_ (black open squares) or *I*_Na(L)_ (black filled squares) observed in GH_3_ cells (mean ± SEM; *n* = 8–9 for each point). The least-squares fit to the Hill equation is represented by the sigmoidal blue or red curve. The IC_50_ value for the TPM-mediated block of *I*_Na(T)_ or *I*_Na(L)_ identified in these cells was 22.4 and 4.1 µM, respectively. The statistical analyses were made by ANOVA-2 for repeated measures, *p* (factor 1) < 0.05, *p* (factor 2) < 0.05, *p* (interaction) < 0.05, followed by post-hoc Fisher’s LSD test, *p* < 0.05

In the first stage of measurements, we investigated whether exposure of cells to TPM could modify the density of peak or transient *I*_Na_ (*I*_Na(T)_) and sustained or late *I*_Na_ (*I*_Na(L)_) in these cells. To do this, we placed cells in Ca^2+^-free, Tyrode’s solution containing 10 mM TEA and 0.5 mM CdCl_2_, and the measuring electrode was filled with a Cs^+^-containing solution. The presence of TEA and CdCl_2_ in the extracellular solution was used to block K^+^ and Ca^2+^ currents, respectively, to isolate the *I*_Na_ current. The voltage-clamp protocol applied (as indicated in the upper part of Fig. 1A) comprised a pre-test pulse from − 80 to − 100 mV for 40 ms to ensure complete recovery of *I*_Na_; a depolarizing step to − 10 mV was given to evoke *I*_Na_, and a return to − 30 mV for a duration of 40 ms was then applied to measure tail component of *I*_Na_. As demonstrated in Fig. 1A, one minute after cell exposure to 10 or 30 µM TPM, the densities of *I*_Na(T)_ and *I*_Na(L)_ measured at the beginning and end-pulse of the depolarization step were progressively diminished. For example, when exposed to concentrations of 10 or 30 µM of TPM, respectively, there was a significant reduction in both *I*_Na(T)_ and *I*_Na(L)_ densities. Specifically, at 10 µM TPM, the densities decreased drastically to 17.5 ± 1.7 pA/pF (*n* = 8, paired *t*-test, *p* < 0.05) for *I*_Na(T)_ and 0.6 ± 0.1 pA/pF (*n* = 8, paired *t*-test, *p* < 0.05) for *I*_Na(L)_. Similarly, exposure to 30 µM TPM resulted in densities of 5.8 ± 0.9 pA/pF (*n* = 8, paired *t*-test, *p* < 0.5) for *I*_Na(T)_ and 0.3 ± 0.1 pA/pF (*n* = 8, paired *t*-test, *p* < 0.05) for *I*_Na(L)_, significantly lower than the control values of 23.1 ± 1.8 pA/pF (*n* = 8) and 0.8 ± 0.3 pA/pF (*n* = 8). Upon removal of TPM, *I*_Na(T)_ and *I*_Na(L)_ densities returned to 23.9 ± 2.1 pA/pF (*n* = 8) and 0.8 ± 0.3 pA/pF (*n* = 8), respectively. Furthermore, the time constant in the slow component of *I*_Na(T)_ inactivation (τ_inact(S)_) with the presence of 10 or 30 µM TPM was respectively shortened to 3.1 ± 0.9 ms (*n* = 8, paired *t*-test, *p* < 0.05) or 1.9 ± 0.7 ms (*n* = 8, paired *t*-test, *p* < 0.05) from a control value of 5.2 ± 1.1 ms (*n* = 8); however, the value of the fast component of current inactivation remained unchanged.

We further constructed the relationship between the TPM concentration and the percentage decrease of *I*_Na(T)_ or *I*_Na(L)_ in GH_3_ cells (Fig. 1B). The addition of TPM to the bath caused a concentration-dependent decrease in the density of either *I*_Na(T)_ or *I*_Na(L)_. We used a Hill function to fit the experimental data and obtained effective IC_50_ values of 22.4 µM and 4.1 µM for TPM-mediated block of *I*_Na(T)_ and *I*_Na(L)_, respectively. Therefore, our experimental results indicate that the TPM exposure produces a concentration-dependent inhibitory effect on both *I*_Na(T)_ and *I*_Na(L)_. Notably, a selective suppression of *I*_Na(L)_ compared to *I*_Na(T)_ was observed, with maximal inhibition of both current occurring at a TPM concentration at approximately 300 µM.

### Effect of TPM on mean current density versus voltage relationship of I_Na(T)_

**Fig. 2:**
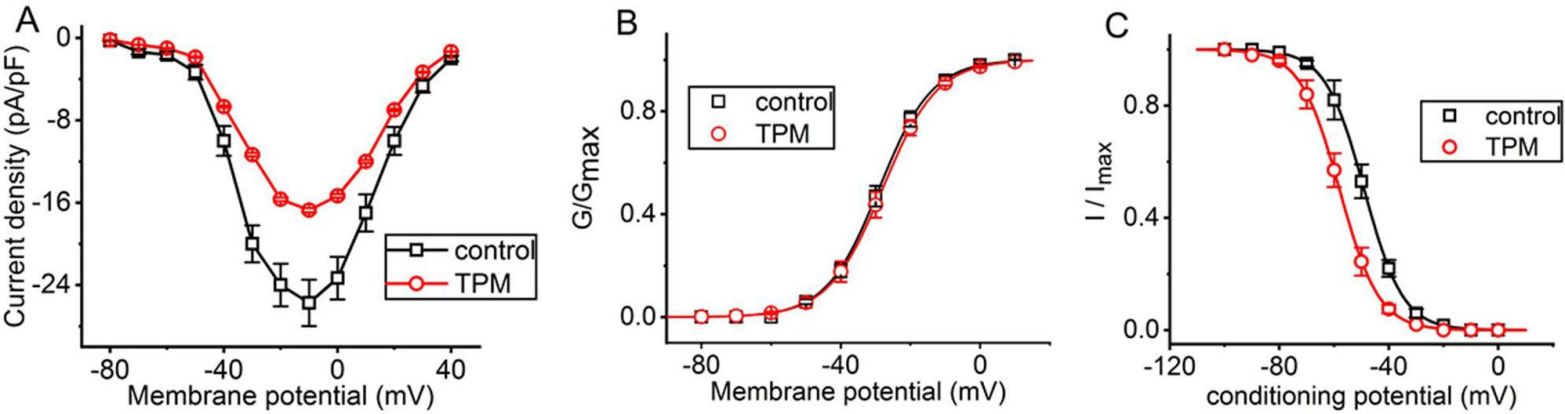
Effect of TPM on the steady-state current density versus voltage relationship (**A**) and the steady-state activation (**B**) or inactivation curve (**C**) of *I*_Na(T)_ in GH_3_ cells. **(A)** Mean current density versus voltage relationships of *I*_Na(T)_ without (black open squares) and with (red open circles) of 10 µM TPM (mean ± SEM; *n* = 8 for each point). Each cell underwent depolarization from − 80 mV to various membrane potentials ranging from − 80 to + 40 mV in 10-mV increments for a duration of 40 ms, with the current density measured at the start of each voltage step. **(B)** and **(C)** respectively illustrate the quasi-steady-state activation and inactivation curves of *I*_Na(T)_ in the absence (black open squares) and presence (red open circles) of 10 µM TPM (mean ± SEM; *n* = 8 for each point). The normalized conductance (i.e., *G*/*G*_max_) or density (i.e., I/I_max_) of *I*_Na(T)_ was plotted against different levels of membrane or conditioning potential. The sigmoidal curves (black and red lines) were fitted to the Boltzmann isotherm using the optimal fit (see Materials and Methods). The statistical analyses in **(A)**, **(B)**, and **(C)** were made by ANOVA-2 for repeated measures, *p* (factor 1) < 0.05, *p* (factor 2) < 0.05, *p* (interaction) < 0.05, followed by post-hoc Fisher’s LSD test, *p* < 0.05

We continued to examine the effects of this drug on *I*_Na(T)_ measured at various membrane potentials. As depicted in Fig. 2A, a steady-state current density versus voltage relationship of *I*_Na(T)_ with or without TPM exposure was further constructed. The application of 10 µM TPM resulted in a striking decrease in the amplitude of *I*_Na(T)_ elicited by a series of voltage steps, especially at the voltages ranging between − 50 and + 10 mV. However, the overall current density versus voltage relationship of *I*_Na(T)_ remained unaltered with TPM exposure, aside from its decrease in current density.

The steady-state activation curve of *I*_Na(T)_ was further determined in the absence and presence of 10 µM TPM, and the results are depicted in Fig. 2B. A smooth sigmoidal curve was generated by fitting the data to the Boltzmann distribution, as outlined in the Materials and Methods section. For both conditions, without and with the application of 10 µM TPM, the midpoint potential (*V*_1/2_) values were found to be − 29 ± 1 mV and − 28 ± 1 mV, respectively (*n* = 8, paired *t*-test, *p* > 0.05). Similarly, the apparent gating charge (*q*) values were 3.3 ± 0.2 *e* and 3.2 ± 0.2 *e*, respectively (*n* = 8, paired *t*-test, *p* > 0.05). Therefore, it can be ascertained that neither *V*_1/2_ nor *q* underwent significant alterations during cell exposure to 10 µM TPM.

### Characterization of TPM-induced modifications on the steady-state inactivation curve of I_Na(T)_

For further evaluation of the TPM-inhibited block of *I*_Na(T)_, we continued to examine the steady-state inactivation curve of the current with a two-step voltage-clamp protocol. Figure 2C demonstrates the steady-state inactivation curve of *I*_Na(T)_ acquired with or without the presence of TPM (10 µM). In this set of experiments, a 40-ms conditioning pulse was given at various membrane potentials to precede the test pulse (40 ms in duration) to − 10 mV from a holding potential of − 80 mV. The relationships between the conditioning potentials and the normalized amplitudes of *I*_Na(T)_ acquired in the control period and with TPM exposure were plotted, and the data were, thereafter, optimally fitted with a single Boltzmann isotherm, detailed under Materials and Methods. The *V*_1/2_ value (i.e., midpoint value) in the control period and during exposure to 10 µM TPM was, respectively, estimated as − 49 ± 3 mV and − 58 ± 3 mV (*n* = 8, *p* < 0.05), whereas the *q* value (i.e., gating charge) in the absence and presence of 10 µM TPM was, respectively, taken to be 3.55 ± 0.09 *e* and 3.52 ± 0.09 *e* (*n* = 8, paired *t*-test, *p* > 0.05). It thus follows that cell exposure to 10 µM TPM not only suppressed the maximal magnitude of *I*_Na(T)_, but it also shifted the quasi-steady-state inactivation curv of *I*_Na(T)_ along the voltage axis to more hyperpolarized potential (i.e., in the leftward direction) by approximately 9 mV. It is therefore reasonable to assume that TPM exposure is capable of diminishing *I*_Na(T)_ magnitude in a voltage-dependent manner in GH_3_ cells.

### Comparative analysis of the impacts of TPM, TPM combined with flumazenil (FLM), and TPM combined with chlorotoxin on I_*Na(T)*_ density

**Fig. 3:**
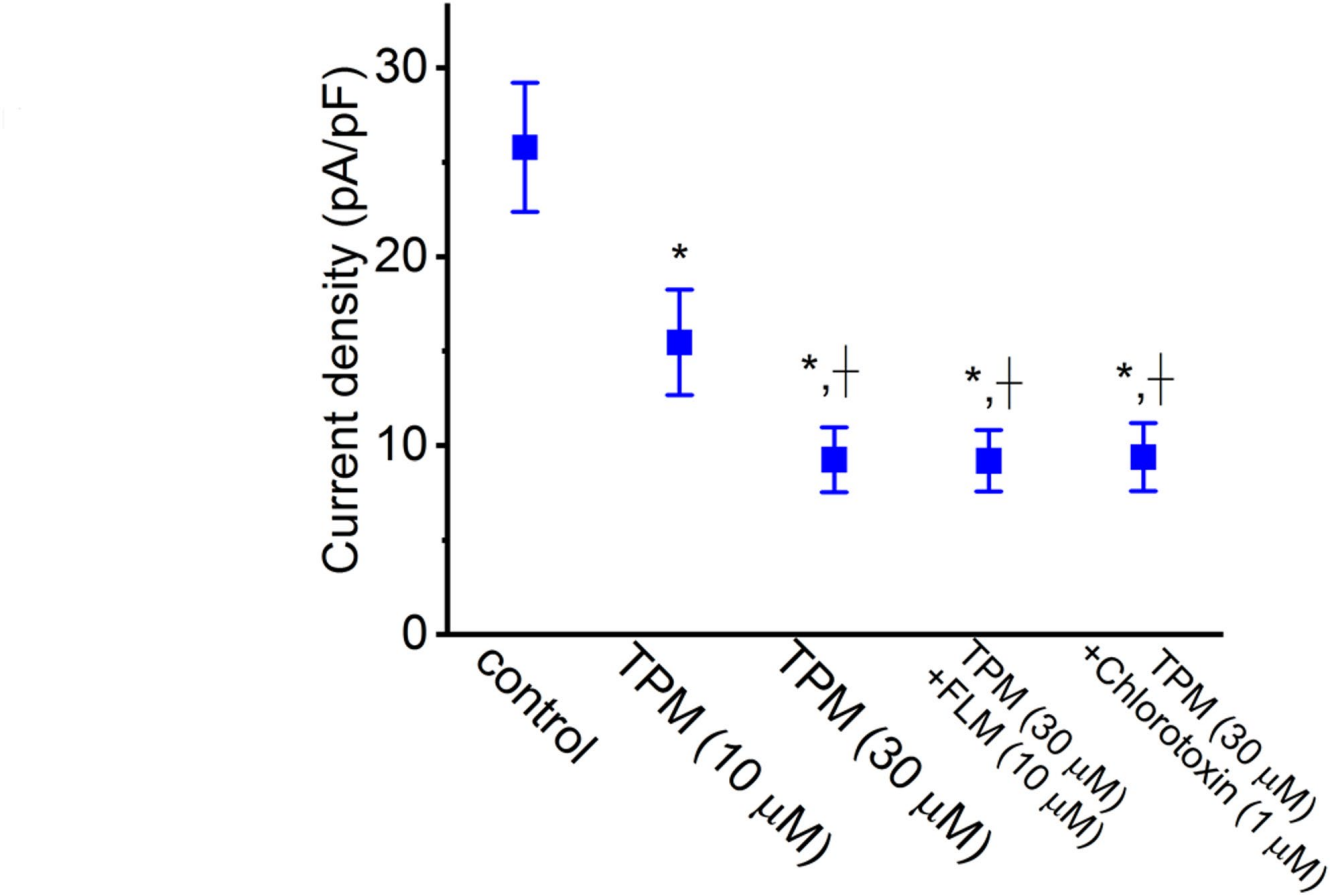
Comparative evaluation of the effects of TPM (10 or 30 µM), TPM in combination with flumazenil (FLM), and TPM in combination with chlorotoxin on the *I*_Na(T)_ density recorded from GH_3_ cells (mean ± SEM; *n* = 8 for each point). The *I*_Na(T)_ density (i.e., absolute value) was measured at the beginning of the 40-ms depolarizing step from − 100 to − 10 mV during cell exposure to different tested compounds. The statistical analyses were made by ANOVA-1, *p* < 0.05, followed by a post-hoc Fisher’s LSD test, *p* < 0.05. ^*^ Significantly different from control (*p* < 0.05), while ^+^ significantly different from TPM (10 µM) alone group (*p* < 0.05)

We next examined if the TPM-induced block of *I*_Na(T)_ could be modified by subsequent addition of FLM or chlorotoxin. FLM is recognized to be an antagonist of the GABA_A_ receptor, while chlorotoxin is a blocker of Cl^−^ channels. As cells were continually exposed to TPM (30 µM), the further application of neither FLM (10 µM) nor chlorotoxin (1 µM) was effective at reversing the TPM-induced block of *I*_Na(T)_ (Fig. 3). We employed a concentration of 30 µM for TPM with the primary objective of ensuring a substantial inhibitory impact on both *I*_Na(L)_ and *I*_Na(T)_. Our simultaneous goal was to explore the potential binding of TPM to GABA_A_ receptors as a mechanism for the inhibition of *I*_Na_. Furthermore, we sought to discern whether the TPM-mediated inhibition of *I*_Na_ is triggered by modifications in chloride currents. The experimental results suggest that the TPM-induced block of *I*_Na(T)_ seen in GH_3_ cells is unlinked to its propensity to interact with GABA_A_ receptors.

### Effect of TPM on hyperpolarization-activated cation current (I_h_) identified from GH_3_ cells

We investigated the biophysical properties of *I*_h_ residing in GH_3_ cells and further studied if the presence of TPM could cause any perturbations on *I*_h_. In this set of experiments, cells were immersed in Ca^2+^-free, Tyrode’s solution, and we filled up the measuring electrode with a K^+^-rich solution. As whole-cell current recordings were established, we clamped the tested cell at − 40 mV and a test pulse from − 40 to − 110 mV for a duration of 2 s was applied to evoke an inward-directed *I*_h_ with the slowly activating time course.

**Fig. 4:**
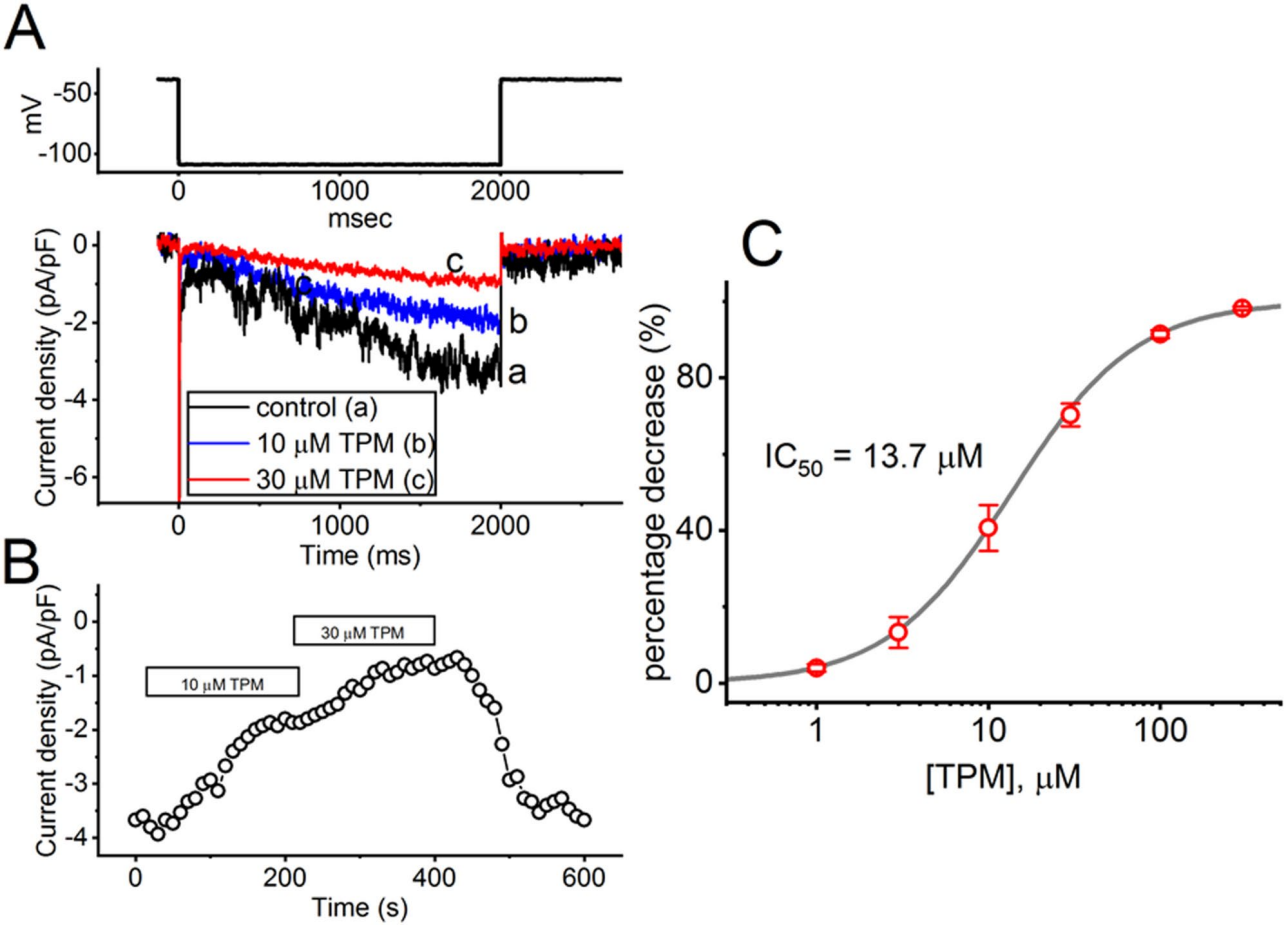
Suppressive effect of TPM on the hyperpolarization-activated cation current (*I*_h_) measured from GH_3_ cells. Cells were placed in Ca^2+^-free, Tyrode’s solution containing 10 mM TEA and 1 µM tetrodotoxin (TTX), while the measuring electrode was filled up with a K^+^-enriched solution. **(A)** Exemplar current traces evoked by a 2-s hyperpolarizing pulse from − 40 to − 110 mV (indicated in the upper part). a: control (i.e., TPM was not present); b: 10 µM TPM; and c: 30 µM TPM. **(B)** Time course depicting the suppressive effect of TPM (10 or 30 µM) on the *I*_h_ density. The current density (indicated in black open circle) was measured at the end of each hyperpolarization step from − 40 to − 110 mV at a rate of 0.1 Hz. The horizontal bar shown above indicates the application of 10 or 30 µM TPM. **(C)** Concentration-dependent inhibitory effect of TPM on *I*_h_ measured from GH_3_ cells. The *I*_h_ densities during different TPM concentrations (1-300 µM) were acquired at the end-pulse of a 1-s hyperpolarizing command voltage from − 40 to − 110 mV. Each point represents the mean ± SEM (*n* = 8–9 for each point). The gray sigmoidal curve drawn was optimally fitted to a Hill equation, as described under Materials and Methods. The statistical analyses were made by ANOVA-1, *p* < 0.05, followed by a post-hoc Fisher’s LSD test, *p* < 0.05

As demonstrated in Fig. 4, one minute after cells were continually exposed to TPM (10 or 30 µM), the *I*_h_ magnitude elicited by the 2-s hyperpolarizing step from − 40 to − 110 mV became progressively decreased. For example, upon the presence of TPM at a concentration of 10 or 30 µM, the *I*_h_ density was vastly decreased to 1.6 ± 0.3 pA/pF (*n* = 8, paired *t*-test, *p* < 0.05) or 0.6 ± 0.1 pA/pF (*n* = 8, paired *t*-test, *p* < 0.05) from a control value of 4.5 ± 0.7 pA/pF (*n* = 8). After the removal of this drug, the current amplitude returned to 4.3 ± 0.6 pA/pF (*n* = 7). Figure 4B illustrates the time course in the suppressive effect of TPM (10 or 30 µM) on the *I*_h_ amplitude evoked by the hyperpolarization step. Furthermore, with the presence of 10 or 30 µM, the time constant of *I*_h_ activation (τ_act_) in response to long-lasting hyperpolarization step was prolonged to 445 ± 18 ms (*n* = 8, paired *t*-test, *p* < 0.05) or 592 ± 23 ms (*n* = 8, paired *t*-test, *p* < 0.05), respectively, from a control value of 403 ± 13 ms (*n* = 8).

In the subsequent set of measurements, we explored the impact of TPM dosage on *I*_h_ amplitude in GH_3_ cells. Figure 4C displays that the density of *I*_h_, which was induced by prolonged membrane hyperpolarization, decreased in a concentration-dependent manner upon application of TPM (1-300 µM). Utilizing the Hill equation, we estimated that the IC_50_ value required for the suppressive effect of TPM on *I*_h_ density was 13.7 µM, and, at a concentration of 300 µM, the current density was almost eliminated. Results from these data therefore reflect that this drug has a significant concentration-dependent depressant action on the *I*_h_ density.

### Effect of TPM on the current density versus voltage relationship of I_*h*_ identified from GH_*3*_ cells

**Fig. 5:**
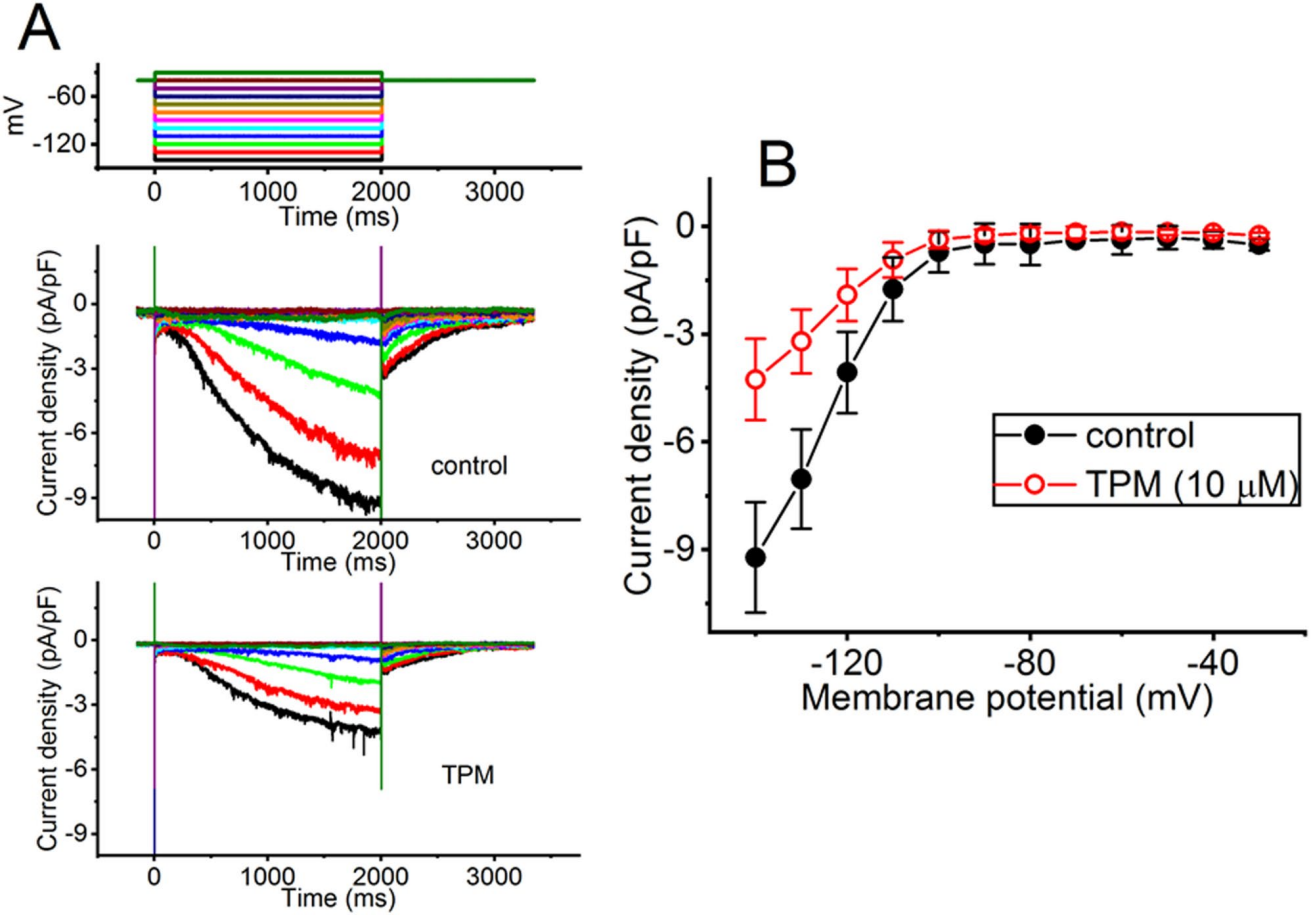
Effect of TPM on mean current density versus voltage relationship of *I*_h_ identified from GH_3_ cells. **(A)** Exemplar current traces obtained during the control period (upper) and with TPM (10 µM) exposure (lower). The voltage protocol applied is illustrated above the current traces. **(B)** Mean current density versus voltage relationship of *I*_h_ without (black filled circles) or with (red open circles) of 10 µM TPM (mean ± SEM; *n* = 8 for each point). The current density was measured at the end-pulse of each voltage step given. The statistical analyses were made by ANOVA-2 for repeated measures, *p* (factor 1) < 0.05, *p* (factor 2) < 0.05, *p* (interaction) < 0.05, followed by post-hoc Fisher’s LSD test, *p* < 0.05

For further characterization of TPM-mediated inhibition of *I*_h_, we additionally examined if cell exposure to TPM could modify any changes in the current density versus voltage relationship of *I*_h_ in these cells. Figure 5A illustrates current traces evoked by the different levels of membrane potential from a holding potential of − 40 mV, as the tested cell was challenged with or without 10 µM TPM. The mean current density versus voltage relationships of *I*_h_ with or without TPM (10 µM) exposure were further constructed in Fig. 5B. As GH_3_ cells were continually exposed to 10 µM TPM, whole-cell conductance of *I*_h_ measured at the voltages ranging between − 140 and − 100 was vastly decreased to 0.11 ± 0.01 nS/pF (*n* = 8, paired *t*-test, *p* < 0.05) from a control value of 0.25 ± 0.01 nS/pF (*n* = 8).

### Suppressive effect of TPM on voltage-dependent hysteresis (Hys_*(V)*_) of I_*h*_ evoked by long-lasting triangular ramp voltage (V_*ramp*_)

**Fig. 6:**
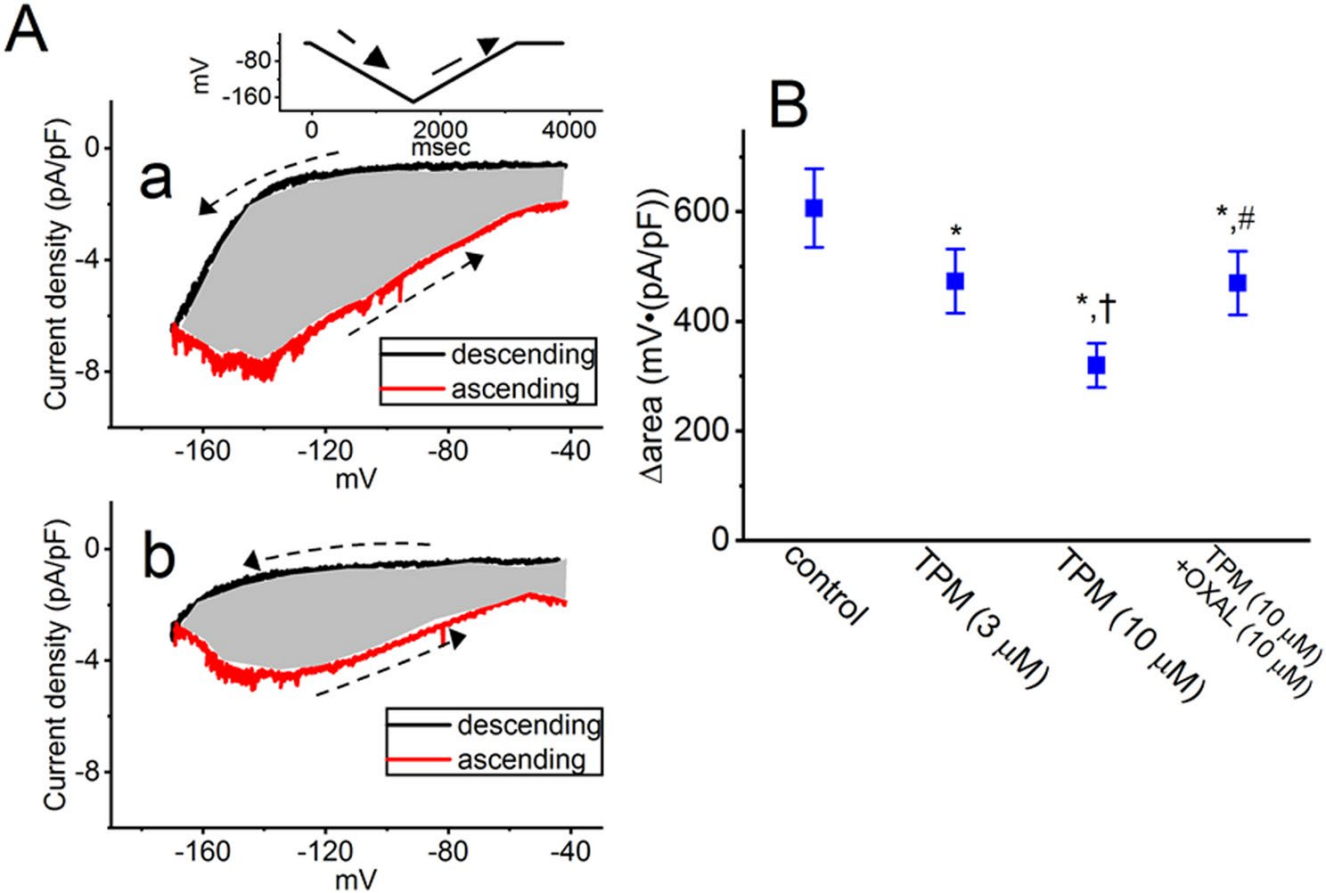
Effect of TPM on the strength in voltage-dependent hysteresis (Hys_(V)_) of *I*_h_ identified from GH_3_ cells. **(A)** Exemplar Hys_(V)_ traces (i.e., the relationship of descending [forward, black color] or ascending [backward, red color] current versus membrane potential of *I*_h_ evoked by the long-lasting inverted double (i.e., inverted isosceles-triangular) ramp voltage (V_ramp_) (indicated in the inset of (Aa)). Hys_(V)_ trace was obtained in the control period (upper, a) and with the presence of 10 µM TPM (lower, b). The dashed arrows along current or potential traces indicate the trajectory with an anticlockwise direction in which time passes with the elicitation by an inverted triangular V_ramp_. The shaded region shows the Hys_(V)_ area (i.e., Δarea) with or without the presence of 10 µM TPM. **(B)** Summary scatter graph demonstrating the effects of TPM (3 or 10 µM) and TPM (10 µM) plus oxaliplatin (OXAL, 10 µM) on the Δarea of *I*_h_’s Hys_(V)_ (i.e., the gray shaded region under the curve activated during the descending and ascending ends of triangular V_ramp_ (mean ± SEM; *n* = 7 for each point). The statistical analyses were made by ANOVA-1, *p* < 0.05, followed by post-hoc Fisher’s LSD test, *p* < 0.05. ^*^ Significantly different from control (*p* < 0.05), ^+^ significantly different from TPM (3 µM) alone group (*p* < 0.05), and ^#^ significantly different from TPM (10 µM) alone group (*p* < 0.05)

Recent studies have revealed the ability of Hys_(V)_ strength of *I*_h_ to regulate different patterns of bursting firing in various types of electrically excitable cells [35–38]. Furthermore, it has been demonstrated that cyclic nucleotide-gated (CNG) channels, including CNGA2 ion channels, exhibit Hys_(V)_ behavior [39]. We hence proceeded to explore whether TPM has the potential to disrupt *I*_h_’s Hys_(V)_ in response to long-lasting triangular V_ramp_. To achieve this, we designed a voltage-clamp protocol and then delivered it to the tested cells. The measurements were carried out on the tested cells clamped at the level of − 40 mV, and we applied a long-lasting inverted V_ramp_ using digital-to-analog conversion to elicit the Hys_(V)_ of *I*_h_. The V_ramp_ protocol consists of a downsloping (forward) limb from − 40 to − 170 mV followed by an upsloping (backward) limb back to − 40 mV. This protocol has a total duration of 3.2 s (i.e., a ramp speed of ± 41 mV/s), as shown in the inset of Fig. 6A. As demonstrated in earlier studies [37, 38, 40], the Hys_(V)_ phenomenon of *I*_h_ (i.e., the relationship of descending or ascending *I*_h_ versus membrane potential) was observed upon activation by a long-lasting inverted triangular V_ramp_, as shown in Fig. 6 [37, 38, 40]. In other words, the *I*_h_ amplitude triggered by the downsloping limb (indicated in black color) of the inverted triangular V_ramp_ was strikingly smaller than that (indicated in red color) by the upsloping end of the V_ramp_. For example, in the control period (i.e., TPM was not present), the *I*_h_ amplitudes at the level of − 140 mV measured during the downsloping and upsloping limbs of triangular V_ramp_ were strikingly distinguishable (i.e., 1.53 ± 0.4 pA/pF [downsloping] versus 7.87 ± 0.7 pA/pF [upsloping]; *n* = 7, paired *t*-test, *p* < 0.05). In particular, upon cell exposure to 10 µM TPM, the *I*_h_ density evoked by the downsloping limb of triangular V_ramp_ was noticed to diminish to a lesser extent than that measured from the upsloping end of V_ramp_. The magnitude of TPM-induced inhibition measured at the downsloping and upsloping limbs of thetriangular V_ramp_ differed significantly. For example, in the presence of 10 µM TPM, the *I*_h_ density at − 140 mV measured from the downsloping and upsloping ends was decreased to 0.8 ± 0.2 pA/pF (*n* = 7, paired *t*-test, *p* < 0.05) and 4.7 ± 0.7 pA/pF (*n* = 7, paired *t*-test, *p* < 0.05), respectively.

Furthermore, the strength (i.e., Δ the area indicated in the shaded region) of *I*_h_ was further quantified with or without the presence of TPM. The experimental observations demonstrated that the amount of Hys_(V)_ responding to the long-lasting V_ramp_ became significantly decreased during cell exposure to TPM (Fig. 6). For example, aside from its suppressive effect on *I*_h_ amplitude, the presence of 10 µM TPM led to a considerable reduction in the *I*_h_’s Δarea responding to inverted triangular V_ramp_, as evidenced by a decrease of Δarea from 606 ± 91 to 321 ± 51 mV·(pA/pF) (*n* = 7, paired *t*-test, *p* < 0.05). After TPM was removed, the Δarea returned to 581 ± 83 mV·(pA/pF) (*n* = 7). In the continued presence of 10 µM TPM, subsequent addition of 10 µM oxaliplatin (OXAL) was able to reverse the *I*_h_’s Δarea to 473 ± 75 mV·(pA/pF) (*n* = 7, paired *t*-test, *p* < 0.05).

### Inhibitory effect of TPM on spontaneous action potentials (APs) measured from GH_3_ cells

**Fig. 7:**
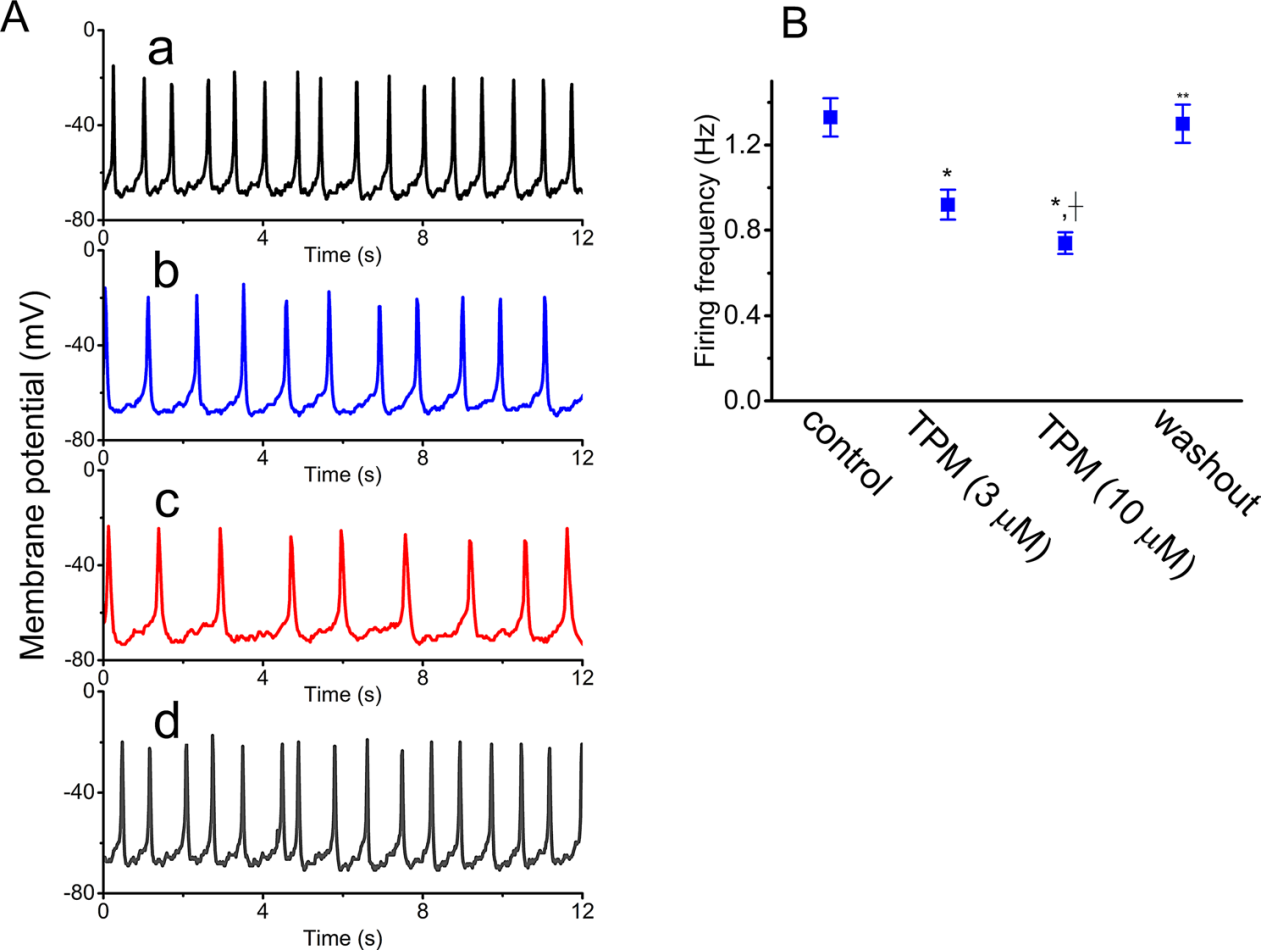
Suppressive effect of TPM on spontaneous action potentials (APs) identified from GH_3_ cells. The whole-cell potential recordings were conducted in cells bathed in normal Tyrode’s solution which contained 1.8 mM CaCl_2_, with the recording electrode filled with K^+^-enriched solution and the current constantly held at 0 pA. **(A)** Exemplar potential traces obtained in the control period (a, TPM was not present, black color), with the presence of 3 µM TPM (b, blue color) or 10 µM TPM (c, red color), and following TPM washout (d, gray color). The upward deflection indicates the appearance of an AP. **(B)** Summary scatter graph demonstrating effect of TPM (3 or 10 µM) on the firing frequency of APs (mean ± SEM; *n* = 8 for each point). The statistical analyses were made by ANOVA-1, *p* < 0.05, followed by post-hoc Fisher’s LSD test, *p* < 0.05. ^*^ Significantly different from control (*p* < 0.05), ^+^ significantly different from TPM (3 µM) alone group (*p* < 0.05), and ^**^ significantly different from TPM (10 µM) alone group (*p* < 0.05)

Based on previous findings that TPM influences sodium-dependent AP firing in mouse spinal cord neurons [41], we conducted a final series of measurements using current-clamp recordings to examine whether TPM modifies spontaneous action potentials. As shown in Fig. 7, the frequency of spontaneous APs gradually decreased within one minute of TPM exposure. Exposure to TPM at a concentration of 10 µM caused a decrease in the firing frequency from 1.33 ± 0.09 to 0.74 ± 0.05 Hz (*n* = 8, paired *t*-test, *p* < 0.05). Concurrently, the cell membrane potential was noted to be hyperpolarized to − 74 ± 2 mV (*n* = 8, paired *t*-test, *p* < 0.05) from a control value of − 69 ± 2 mV (*n* = 8). After TPM was removed, the AP frequency observed under current-clamp conditions returned to 1.30 ± 0.09 Hz (*n* = 8). These results suggest that TPM-mediated changes in ionic currents, specifically *I*_Na_ and *I*_h_, are likely responsible for the reduction in the frequency of spontaneous APs in these cells. This reduction in AP firing frequency may be linked to TPM’s disruption of stimulus-secretion coupling in pituitary cells.

## Discussion

In this investigation, we demonstrate that TPM significantly inhibits *I*_Na(T)_ and *I*_Na(L)_ in pituitary GH_3_ cells. Our results showed that TPM inhibited *I*_Na(L)_ to a greater extent than *I*_Na(T)_ in response to short depolarization steps. The effective IC_50_ values required for TPM-mediated inhibition of *I*_Na(T)_ and *I*_Na(L)_ in these cells were 22.4 and 4.1 µM, respectively. Exposure to 10 µM TPM did not change the overall current density versus voltage relationship of *I*_Na(T)_, but caused a shift of the quasi-steady-state inactivation curve of *I*_Na(T)_ to a more hyperpolarized potential of approximately 11 mV, without altering the gating charge of the curve. This selective inhibition by TPM is of great importance and may contribute to its ability to modulate the electrical properties of various excitable cells, as suggested by previous research [11]. TPM shows a stronger inhibitory effect on the late component of *I*_Na_ than on its peak component. The cause of this difference remains unclear; however, it may be due to TPM’s greater affinity for the inactivated state of Na_V_ channels, rather than for the open state.

Previous studies have demonstrated potential interactions between TPM and GABA_A_ receptors under various experimental conditions [42–47]. Additionally, pituitary cells have been shown to express functional GABA_A_ receptors [48]. However, our findings indicate that the TPM-induced inhibition of *I*_Na(T)_ in GH_3_ cells was not reversed by the application of either FLM, a GABA receptor antagonist, or chlorotoxin, a chloride channel blocker. We observed that TPM effectively suppressed the density of *I*_h_. These findings align with a previous study [49]. Although TPM’s effects on ionic currents (e.g., *I*_Na_ and *I*_h_) appear to be unrelated to its binding to GABA_A_ receptors, further research in this area is still needed in different cell types.

While conducting our experiments, we did not assess the mRNA expression of GABA_A_ receptors or chloride channels. However, when investigating the concentration-dependent effect of TPM on *I*_Na_ amplitude, we concluded that TPM’s impact was direct and independent of any additional binding to different receptors. Additionally, it’s worth noting that we did not introduce GABA into the bath initially, and the GABA level in the bath was expected to be extremely low under these experimental conditions. Future research could explore culturing GH_3_ cells in a TPM-supplemented medium and examining the electrophysiological changes in these cells.

Upon replicating our experiments in the specified conditions, we observed that the reversal potential of *I*_Na_ is approximately + 40 to + 50 mV. One contributing factor is the non-uniformity of the intracellular environment. In the context of whole-cell current recordings during cell dialysis, complete elimination of intracellular Na^+^ ions is unattainable, resulting in the retention of a certain concentration of Na^+^ inside the cell. Another factor could be the potential contamination of additional Na^+^ ions due to other components in our intracellular pipette solution, leading to a slightly elevated concentration. Furthermore, the persistence of residual leak K^+^ current components was also a plausible explanation.

TPM has been demonstrated to be beneficial for the management of migraine pain [3–5], and the magnitude of *I*_h_ in different excitable cells has been noticed to be linked to the occurrence of migraine [29–32]. In addition, TPM has been recently noticed to produce visual deficits [10]. In GH_3_ cells, functional expression of HCN2, HCN3, or both has been observed [24]. However, the presence of heterotetramers or co-expression does not mean that TPM’s blockade of *I*_h_ results exclusively from simultaneously blocking both channels; inhibiting either HCN2 or HCN3 alone might yield similar effects. Additionally, research suggests that CNG channels, which display Hys_(V)_ behavior, play a role in visual receptor potentials [39], and recent studies have demonstrated functional HCN channels in vestibular ganglion neurons [50]. These findings imply that alterations in HCN channel activity are crucial for determining the timing and sensitivity of afferent responses. Further investigation is needed to assess whether TPM’s blockade of *I*_h_ contributes to the visual deficits or vestibular dysfunction observed in treated individuals [10, 50].

The *I*_h_ current elicited by a triangular V_ramp_ disrupts the electrical behavior of excitable cells by influencing voltage-sensing domain relaxation and Hys_(V)_ in channel proteins, particularly HCNx channels [34–52]. These channels also display a form of “inertia” similar to the Hys(V) observed in ferromagnetic materials [53, 54]. In GH_3_ cells, *I*_h_ exhibits a non-equilibrium, anti-clockwise Hys_(V)_ loop in response to an isosceles-triangular V_ramp_, linked to state-dependent changes in the voltage sensitivity of gating charge movements [34, 38, 40, 51, 52]. Exposure to TPM significantly reduced Hys_(V_), an effect that was reversed by OXAL. These findings suggest that TPM may interact with the voltage-sensing domains of HCNx channels, which could have implications for clinical efficacy and adverse effects, particularly in combination therapies.

Multiple studies have linked migraine pathophysiology to HCNx channels, particularly HCN2 [29–32]. CNG and HCN channels also play a role in phototransduction in photosensitive retinal ganglion cells [33]. Furthermore, human research has implicated HCN2 in juvenile myoclonic epilepsy [55], and HCN2 knockout animals exhibit spontaneous absence seizures and cardiac sinus arrhythmia [56, 57]. Elevated *I*_h_ may also contribute to persistent dendritic hyperexcitability in the hippocampus following febrile seizures, challenging its traditionally viewed anti-convulsive role [58]. Therefore, TPM’s ability to suppress *I*_h_, as observed in our study, likely contributes to its efficacy in alleviating migraine pain and epileptic seizures.

*I*_h_ h acts as a shunt to regulate the amplitude and duration of excitatory postsynaptic potentials and to limit temporal summation [59]. Human studies suggest that HCN2 contributes to juvenile myoclonic epilepsy [56], and its prominent expression in the thalamus facilitates the spontaneous firing of thalamocortical neurons during oscillations and 3-Hz spike-and-wave patterns [55]. In a pilocarpine-induced epilepsy model, dendritic HCN1 and HCN2 channels were initially downregulated but later increased during the chronic phase [60], indicating that regional *I*_h_ density may influence seizure susceptibility or resistance. Additionally, elevated *I*_h_ in the hippocampus after febrile seizures may promote persistent dendritic hyperexcitability, challenging its traditionally anti-convulsive role [58]. Given TPM’s established efficacy in treating both generalized and focal epilepsy [61], further studies are needed to clarify its combined effects on *I*_h_ and *I*_Na_ in epileptogenesis.

In this study, it was found that the IC_50_ values required for TPM to block *I*_Na(T)_ and *I*_Na(L)_ were 22.4 and 4.1 µM, respectively. These values are lower than the ones reported for the TPM-mediated block of *I*_Na_ in cerebellar granule cells [19] or Na_V_1.3a-expressing HEK293 cells [20]. The mRNA transcripts for the α-subunit of Na_V_1.1, Na_V_1.2, and Na_V_1.6 were reported to be expressed in pituitary GH_3_ cells [62]. In addition, TPM exposure caused a decrease in *I*_h_ density, with an IC_50_ value of 13.7 µM. The TPM’s ability to suppress the firing of spontaneous APs was also observed under current-clamp conditions. Furthermore, the blood level during intravenous administration of TPM was reported to range between 6 and 12 µM; therefore, effects of TPM on these channels could be clinically achievable [63].

Whether the outcomes of this experiment in our study extend to other types of excitable cells, such as primary nerve cells and cardiac cells, necessitates further investigation in the future. Abnormalities, whether gain or loss of function, in different ion channels within electrically excitable cells are closely linked to various neurological disorders, including epileptic disorders. These ion currents are also present in various neuroendocrine or endocrine cells [62]. Consequently, influencing these currents will impact stimulus-secretion coupling, thereby influencing the release of hormones of different types.

In conclusion, our study has unveiled that TPM exerts a time-, concentration-, and voltage-dependent suppression of *I*_Na_. Moreover, TPM was observed to alter the magnitude, gating properties, and Hys_(V)_ behaviors of *I*_h_. Additionally, exposure to TPM resulted in a reduction in the frequency of spontaneous action potentials. It’s worth noting that the impact of TPM on excitable cells may fluctuate based on factors such as the resting membrane potential, firing patterns, and the concentrations of TPM administered. Previous research has suggested that TPM may cause arrhythmic side effects at the concentrations used in this study [64]. These discoveries prompt us to suggest that the simultaneous inhibition of both *I*_Na_ and *I*_h_ by TPM, as witnessed in our investigation, could potentially have implications for the clinical and therapeutic use of this medication. Given that the modulation of Na_V_ or HCN channels by TPM seems to be intricately linked to specific pathways and receptors, additional research is imperative to further explore this complexity.

## Data Availability

The datasets used and/or analyzed during the current study are available from the corresponding author on reasonable request.

## Abbreviations

ANOVA: Analysis of variance
AP: Action potential
CNG channel: Cyclic nucleotide-gated channel
FLM: Flumazenil
GABA: γ-aminobutyric acid
GABA_A_ receptor: γ-aminobutyric acid type A receptor
HCN channel: Hyperpolarization-activated cyclic nucleotide-gated channel
Hys_(V)_: Voltage-dependent hysteresis
IC_50_: Concentration required for 50% inhibition
I_h_: Hyperpolarization-activated cation current
I_Na_: Voltage-gated Na^+^ current
I_Na(L)_: Late Na^+^ current
I_Na(T)_: Transient Na^+^ current
LSD test: Least-significant difference test
Na_V_ channel: Voltage-gated Na^+^ channel
OXAL: Oxaliplatin
SEM: Standard error of mean
τ_act_: Activation time constant
τ_inact(S)_: Time constant in the slow component of I_Na(T)_ inactivation
TEA: Tetraethylammonium chloride
TPM: Topiramate (Topamax^®^)
TTX: Tetrodotoxin
V_ramp_: ramp voltage
